# Pediatric Urological Emergencies at a Regional Hospital in Burkina Faso: A Series of 28 Cases

**DOI:** 10.1155/aiu/1195422

**Published:** 2026-06-30

**Authors:** Hassami Sawadogo, Abdoul-Karim Paré, Abdoul-Karim Ouattara, Fatao Ouédraogo, Békaye Ouédraogo, Tiéoulé Mamadou Traoré, Clotaire Alexis Marie Kiemdiba Donega Yaméogo, Brahima Kirakoya, Adama Ouattara

**Affiliations:** ^1^ Department of Urology, Dedougou Regional Hospital, Dedougou, Burkina Faso; ^2^ Division of Urology, Souro Sanou University Teaching Hospital, Bobo-Dioulasso, Burkina Faso; ^3^ Division of Chirurgy, Manga Regional Hospital, Manga, Burkina Faso; ^4^ Division of Urology, Yalgado Ouedraogo University Teaching Hospital, Ouagadougou, Burkina Faso; ^5^ Division of Urology, Ouahigouya University Teaching Hospital, Ouahigouya, Burkina Faso

**Keywords:** Burkina Faso, pediatrics, scrotal trauma, testicular torsion, urological emergency

## Abstract

**Background:**

Pediatric urological emergencies are potentially serious conditions that can compromise functional or even vital prognosis if not managed promptly. In sub‐Saharan Africa, their true incidence and management modalities remain poorly documented, particularly in regional‐level hospitals. This study aims to describe the epidemiological, clinical, therapeutic, and outcome aspects of pediatric urological emergencies managed at the Regional Hospital Center of Dédougou, Burkina Faso.

**Patients and Methods:**

A retrospective descriptive study was conducted from April 1, 2025, to March 31, 2026, in the surgery department of the Regional Hospital Center of Dédougou. All children aged 0 to 15 years admitted for a urological emergency and who received specialized care were included.

**Results:**

Twenty‐eight (28) patients were identified, with a mean age of 8.22 years (range: 0–15 years) and a male‐to‐female ratio of 27:1. Traumatic emergencies accounted for 42.85% of cases (*n* = 12), dominated by scrotal trauma (*n* = 6), including three cases of bovine goring. Functional and vascular emergencies represented 35.7% (*n* = 10), mainly testicular torsions (*n* = 6) with a mean consultation delay of 65.3 h and an orchiectomy rate of 66.7% (4/6). Obstructive emergencies accounted for 17.85% (*n* = 5). Overall outcomes were favorable in all 28 patients, with no mortality recorded.

**Conclusion:**

In our setting, pediatric urological emergencies are dominated by trauma and are characterized by significant consultation delays. Strengthening community awareness, healthcare worker training, and regulation of traditional practices is essential to improve functional prognosis.

## 1. Background

Pediatric urological emergencies encompass a heterogeneous group of clinical situations whose rapid assessment and management are essential to preserve urological health and prevent irreversible functional or vital complications [1, 2]. They may be congenital, traumatic, infectious, obstructive, or iatrogenic in origin. In children, the anatomical and physiological peculiarities of the urogenital tract, combined with specific sociocultural factors, can lead to atypical presentations and diagnostic delays [3].

In low‐resource countries, particularly in sub‐Saharan Africa, these emergencies are often marked by delayed consultation, insufficient specialized facilities, and the persistence of potentially harmful traditional practices, especially during circumcision [4, 5]. These factors contribute to high morbidity, which may result in organ loss or permanent functional sequelae.

Several African studies report a predominance of external genital trauma and neglected testicular torsions among pediatric urological emergencies [6, 7]. In Senegal, Sarr et al. reported a mean consultation delay of 102 h for testicular torsions [7].

However, data from regional hospitals, which often serve as the first level of specialized referral care, remain scarce in the literature. The Regional Hospital Center (CHR) of Dédougou, located in the Bankui region in Northwestern Burkina Faso, serves a predominantly rural population engaged mainly in agropastoral activities. The aim of this study was to describe our department’s experience in managing pediatric urological emergencies over a one‐year period, to contribute to a better understanding of this condition in our context, and to identify areas for improvement.

## 2. Patients and Methods

This was a retrospective descriptive study conducted in the surgery department of the CHR of Dédougou. The CHR of Dédougou is a second‐level referral hospital in the Bankui region, serving a population of approximately 1.8 million inhabitants, mostly rural. The study period spanned from April 1, 2025, to March 31, 2026 (12 consecutive months). Patients included in the study were children aged 0 to 15 years admitted for a urological emergency, with a diagnosed urological emergency and complete, usable medical records. Patients were excluded if their medical records were incomplete or if they had nonurgent urological conditions. During the study period, a total of 33 children were initially identified with a urological emergency. Five were excluded due to incomplete or missing medical records, leaving 28 patients for analysis. Data were extracted from medical records, consultation registers, operative reports, and hospitalization registers. The following variables were recorded: patient age and sex, circumstances of occurrence, consultation delay, final diagnosis, therapeutic management, and clinical outcome.

## 3. Results

### 3.1. Epidemiological Aspects

During the study period, 28 children were managed for urological emergencies. The mean age was 8.22 ± 4.3 years (median: 9 years; range: 0–15 years). Age distribution showed a predominance of children aged 6–10 years (*n* = 12; 42.9%), followed by 11–15 years (*n* = 7; 25%) and 0–5 years (*n* = 9; 32.1%). The male‐to‐female ratio was 27:1, with 27 boys (96.4%) and 1 girl (3.6%).

Most patients came from rural areas (*n* = 22; 78.6%) versus 6 (21.4%) from urban areas. The mean consultation delay was 38.6 ± 32.4 h (median: 24 h; range: 2–168 h). This delay exceeded 24 h in 53.57% of cases (*n* = 15). Detailed demographic characteristics are presented in Table 1.

**TABLE 1:** Demographic characteristics of patients (*n* = 28).

Variable	Number (*n*)	Percentage (%)
Age group		
0–5 years	9	32.1
6–10 years	12	42.9
11–15 years	7	25
Sex		
Male	27	96.4
Female	1	3.6
Origin		
Rural	22	78.6
Urban	6	21.4

### 3.2. Distribution of Urological Emergencies

**FIGURE 1:**
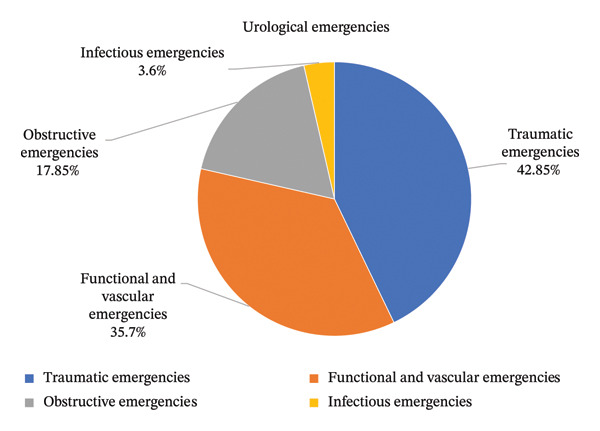
Distribution of urological emergencies according to pathophysiological mechanism/context of occurrence.

Pediatric urological emergencies were classified into four main groups based on pathophysiological mechanism: traumatic (*n* = 12; 42.85%), functional and vascular (*n* = 10; 35.7%), obstructive (*n* = 5; 17.85%), and infectious (*n* = 1; 3.6%). The distribution is illustrated in Figure 1.

#### 3.2.1. Traumatic Emergencies (*n* = 12; 42.85%)

**FIGURE 2:**
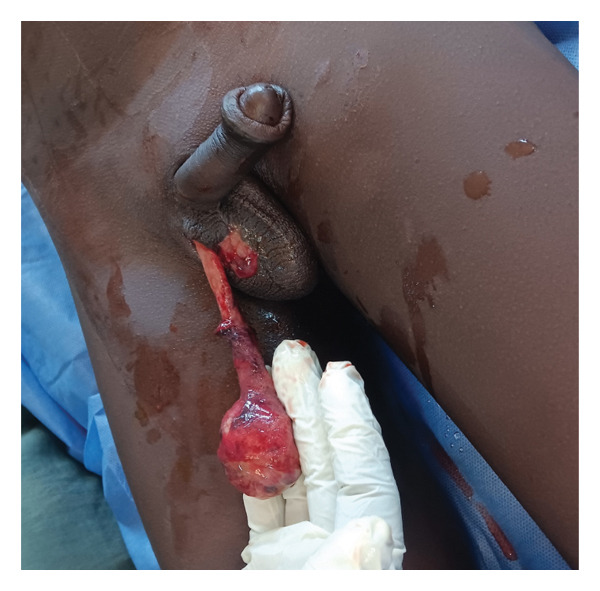
Bovine goring of the scrotum with exteriorization of the right testis and spermatic cord. Note the hematoma of the spermatic cord.

**FIGURE 3:**
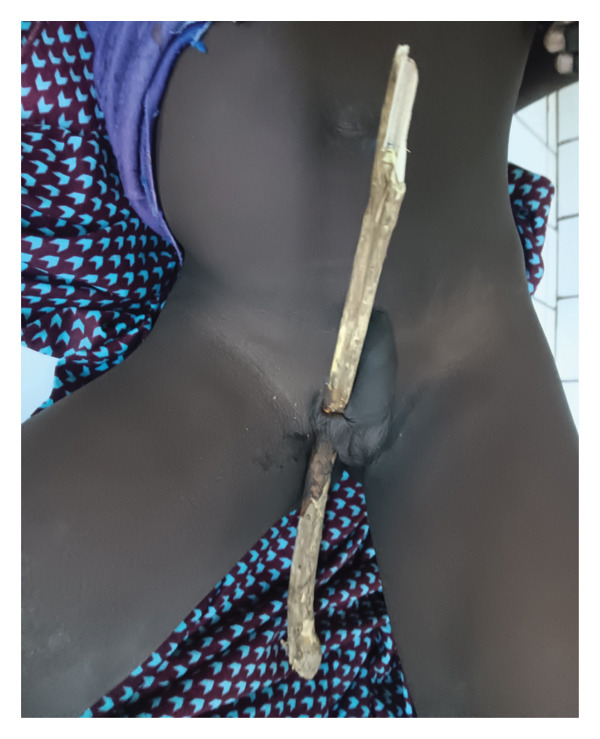
Impalement of the right scrotum by a dry wooden stick.

Scrotal trauma predominated (*n* = 6), including three cases of bovine goring (Figure 2) affecting children aged 8, 10, and 12 years who were entrusted with cattle herding in rural agropastoral areas, one case of impalement by dry wood during a fall on a football field (Figure 3), one school fight–related scrotal bite, and one open scrotal wound from a bicycle fall. All patients underwent the required surgical exploration and debridement and received tetanus prophylaxis combining tetanus toxoid vaccine and tetanus immunoglobulin. All three bovine goring cases underwent scrotal reconstruction and received antibiotic prophylaxis (amoxicillin–clavulanic acid + metronidazole); anti‐rabies post‐exposure prophylaxis was not administered as the injuries were inflicted by cattle, which are not considered a rabies vector species in our context. Testicular preservation rate was 100% in this subgroup.

**FIGURE 4:**
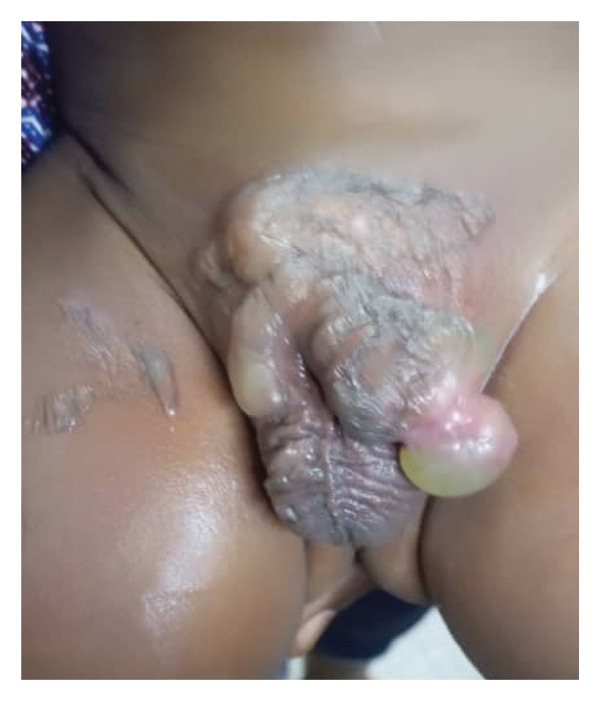
Deep second‐degree burn of the external genitalia in a 24‐month‐old infant.

**FIGURE 5:**
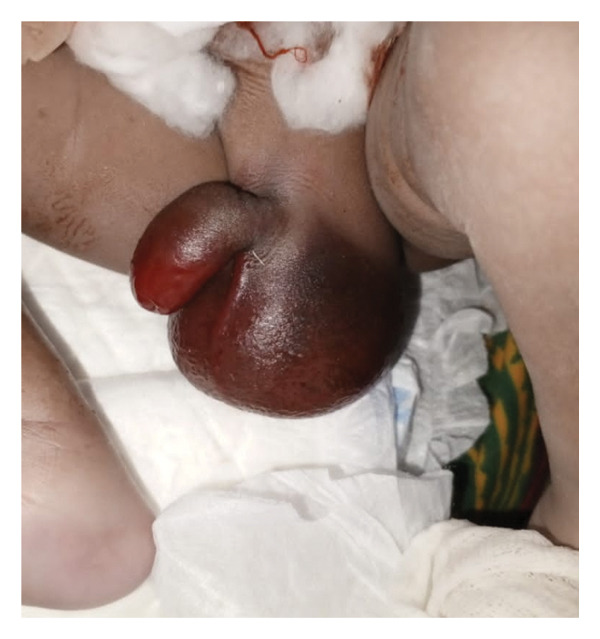
Neonatal penoscrotal ecchymosis and edema following cesarean delivery.

**FIGURE 6:**
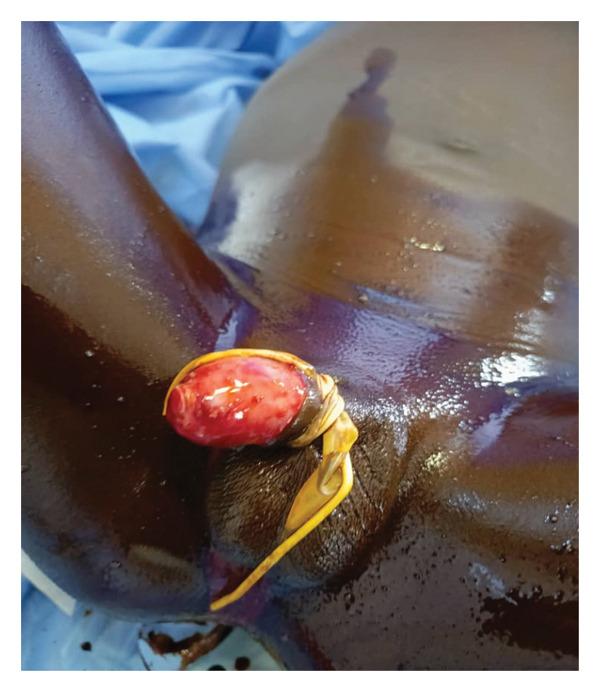
Preoperative image of accidental complete section of the distal third of the penis during circumcision.

Two cases of second‐degree genital burns (one flame burn and one scald burn from hot liquid) (Figure 4), two cases of neonatal penoscrotal trauma, one during a vaginal delivery and the other by cesarean section (Figure 5), one case of closed kidney trauma on preexisting ureteropelvic junction obstruction (UPJO) (which manifested as lumbar pain associated with hematuria following blunt abdominal trauma during a bicycle fall), and one case of iatrogenic amputation of the distal third of the penis during a circumcision (Figure 6) were also observed.

All these cases were managed with favorable outcomes: the genital burns received temporary urethral catheterization, regular local dressings, antibiotic prophylaxis, and analgesia (paracetamol 15 mg/kg every 6 h combined with ibuprofen 10 mg/kg every 8 h for 7 to 10 days) with complete healing and no urethral stenosis. The neonatal traumas were treated medically with corticosteroids after ultrasound confirmation of the absence of hematocele or testicular injury. The closed renal trauma was initially managed conservatively (bed rest and urethral catheterization) followed by delayed open pyeloplasty at 2 months; the affected kidney was preserved, with normal postoperative renal function at the 9‐month follow‐up visit. The penile amputation underwent successful reimplantation within 3 h without loupes, with preserved urinary stream and glans sensitivity at 9‐month follow‐up.

#### 3.2.2. Functional and Vascular Emergencies (*n* = 10; 35.7%)

**FIGURE 7:**
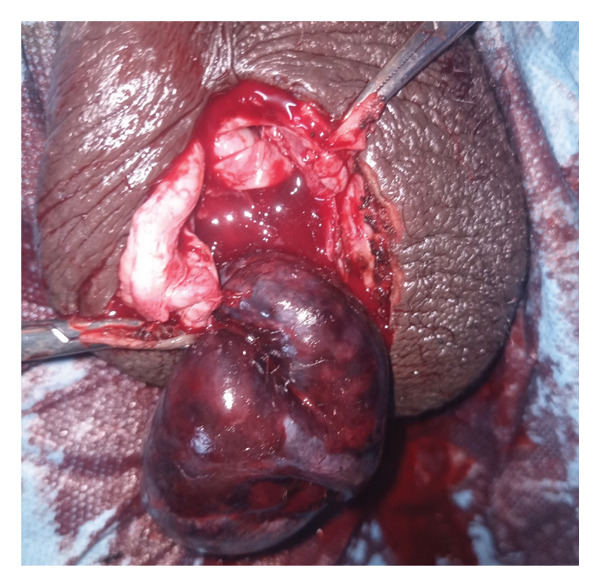
Intraoperative image of neglected right testicular torsion showing complete testicular necrosis.

Testicular torsions accounted for 60% (*n* = 6), with mean age of 11.5 ± 2.8 years (range: 8–15). All patients presented after > 24 h (mean delay: 65.3 ± 28.4 h; median: 63 h; range: 30–120). Surgical exploration revealed necrosis in 4 cases (66.7%; Figure 7), requiring orchiectomy (contralateral orchidopexy delayed 1 month). In 2 cases (33.3%), the testis was viable, allowing detorsion and bilateral orchidopexy.

**FIGURE 8:**
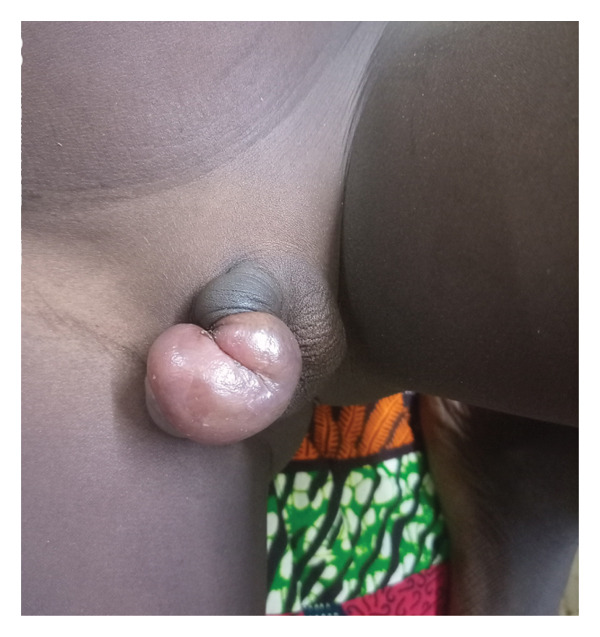
Preoperative image of iatrogenic paraphimosis in an uncircumcised child.

**FIGURE 9:**
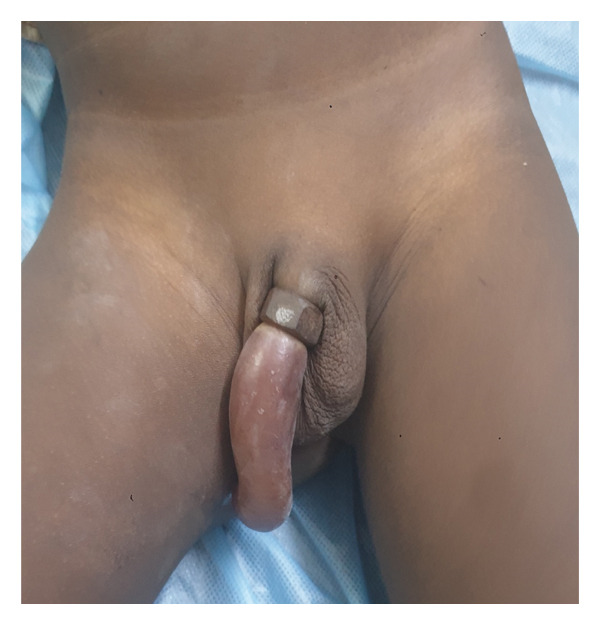
Penile strangulation by a metallic nut inserted voluntarily in a playful context in a 7‐year‐old boy.

Three paraphimosis cases (two of which were iatrogenic after catheterization) (Figure 8) and one case of penile strangulation by a metal nut (Figure 9) were successfully treated conservatively.

#### 3.2.3. Obstructive Urological Emergencies (*n* = 5; 17.85%)

**FIGURE 10:**
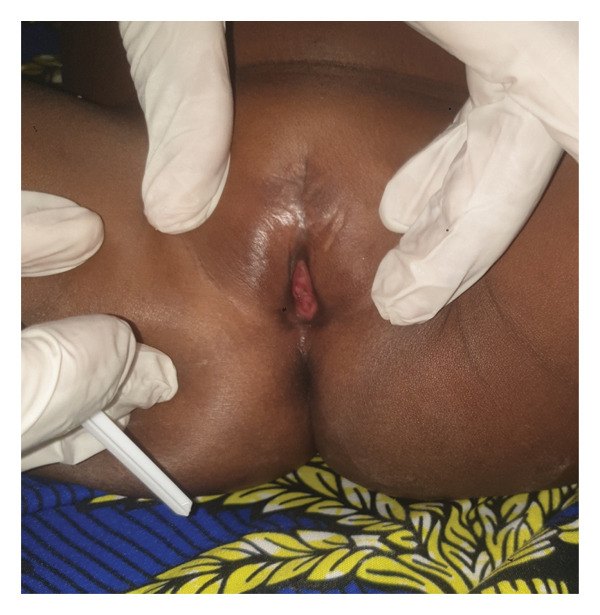
Genital appearance of a 4‐year‐old girl who underwent Type III female genital mutilation, presenting with meatal stenosis and chronic urinary retention.

Five cases of obstructive emergencies were treated. Two children (aged 4 and 6 years) presented with acute urinary retention due to bladder stones measuring 18 mm and 22 mm on ultrasound; both underwent emergency urethral catheterization followed by open cystolithotomy. A 15‐year‐old boy was admitted for renal colic secondary to a 23‐mm pelvic stone (confirmed by noncontrast CT); initial medical analgesia was followed by delayed pyelolithotomy at 1 month. A 4‐year‐old boy presented with complete urinary retention due to an impacted urethral stone; meatotomy allowed stone extraction and immediate resumption of spontaneous voiding. Finally, a 4‐year‐old girl had chronic urinary retention caused by meatal stenosis secondary to Type III female genital mutilation (Figure 10); progressive urethral dilation using bougies resulted in satisfactory urination without post‐void residual urine. Stone composition analysis was not performed in any of the four urolithiasis cases, as our institution lacks a biochemical stone analysis laboratory. Infrared spectroscopy or wet chemical analysis was therefore not available. This represents a limitation of our study.

#### 3.2.4. Infectious Emergencies (*n* = 1; 3.6%)

An 8‐year‐old boy (3.6%) consulted for fever (temperature 38.9°C), intense local pain, and purulent discharge at the circumcision wound site, prompting emergency consultation five days after the procedure. He presented with a surgical site infection following a circumcision performed at a peripheral health center. A pus swab isolated *Staphylococcus aureus* sensitive to oxacillin, amoxicillin/clavulanic acid, and fusidic acid. He received injectable antibiotic therapy and ointment, along with daily dressing changes. His condition improved.

Table 2 summarizes the different pediatric urological emergencies encountered and the treatments performed.

**TABLE 2:** Summary of pediatric urological emergencies.

Emergency group	Number	Pathologies	Treatments
Traumatic emergencies	12 (42.85%)	Scrotal goring by cattle (3)	Debridement and scrotal repair
		Burns of external genitalia (2)	Dressings, urinary catheterization
		Bicycle fall with scrotal wound (1)	Debridement and scrotal repair
		Neonatal trauma of external genitalia (2)	Medical treatment with corticosteroid therapy
		Complete penile section during circumcision (1)	Successful penile reimplantation
		Scrotal bite during school fight (1)	Debridement and scrotal repair
		Scrotal impalement by dry wood (1)	Debridement and scrotal repair
		Blunt renal trauma on UPJO (1)	Urinary catheterization, bed rest, delayed pyeloplasty

Functional and vascular emergencies	10 (35.7%)	Neglected testicular torsions (6)	Orchiectomy for necrosis in 4 cases; orchidopexy
		Penile strangulation by metal nut (1)	Disincarceration after manual reduction of edema
		Paraphimosis (3)	Manual reduction followed by circumcision

Obstructive urinary emergencies	5 (17.85%)	Acute urinary retention due to bladder stones (2)	Urinary catheterization followed by cystolithotomy
		Renal colic due to pelvic stone (1)	Medical treatment with ketoprofen followed by delayed pyelolithotomy
		Chronic urinary retention due to FGM sequelae (1)	Progressive urethral dilations
		Complete urinary retention due to impacted urethral stone (1)	Meatotomy + extraction of the stone

Infectious emergencies	1 (3.6%)	Surgical site infection after circumcision (1)	Antibiotics, dressings
Total	28 (100%)		

### 3.3. Outcomes and Complications

Mean hospital stay was 2.8 days. Short‐term outcome (mean follow‐up 8.3 months) was favorable in 100% (*n* = 28). No deaths occurred.

For testicular torsions, testicular salvage rate was 33.3% (2/6), corresponding to the shortest delays (30 and 36 h). The orchiectomy rate was 66.7% (4/6), all cases involving patients who consulted after 48 h.

## 4. Discussion

Our study reports 28 cases of pediatric urological emergencies managed over one year at the CHR of Dédougou. The male predominance (sex ratio: 27:1) and mean age of 8.22 years are consistent with African literature data [6, 7]. The predominantly rural origin of patients (78.6%) reflects the hospital’s catchment area and partly explains the specific pattern of emergencies encountered, particularly trauma related to agropastoral activities. As shown in Figure 1, traumatic conditions predominated (42.85%), followed by functional and vascular emergencies (35.7%).

The mean consultation delay of 38.6 h, with 53.57% of patients presenting after 24 h, represents the major issue in our series and directly impacts functional prognosis. For testicular torsions, the mean delay of 65.3 h far exceeds recommendations (< 6 h) [8, 9]. Similar findings have been reported in Senegal (102 h) [7] and in a review in sub‐Saharan Africa (52.5 h) [10]. Geographical distance, economic constraints, low community awareness, and frequent recourse to traditional medicine explain these delays [11]. The consequences are dramatic: our 66.7% orchiectomy rate for testicular torsion contrasts with the 20%–40% rates reported in developed countries [8]. Importantly, the two preserved testes in our series corresponded to the shortest consultation delays (30 and 36 h), confirming the critical importance of early management. This finding underscores the urgent need for targeted awareness campaigns and strengthened referral pathways.

The predominance of traumatic emergencies (42.85%), mainly scrotal trauma, is characteristic of our rural setting. The three cases of bovine goring (10.71% of the entire series) represent a remarkable specificity linked to children being entrusted with cattle herding in agropastoral communities. All scrotal trauma cases underwent systematic surgical exploration with a 100% testicular preservation rate, confirming the value of prompt and systematic exploration as recommended [12, 13].

The case of penile amputation during circumcision by paramedical personnel illustrates a recurrent problem in sub‐Saharan Africa. Major circumcision complications include hemorrhage, infection, partial or complete penile amputation, meatal stenosis, and urethrocutaneous fistulae [14, 15]. Successful reimplantation of the glans in our case, despite the absence of microsurgical equipment, demonstrates that good outcomes are possible with limited resources when management is prompt. Literature reports high success rates for penile replantation, with satisfactory outcomes in over 90% of cases when performed promptly [16]. In our case, the 3‐hour delay and preservation of the amputated segment in a cool environment likely contributed to the success.

The two iatrogenic paraphimosis cases post‐catheterization raise important medicolegal and public health concerns. These preventable complications, together with the penile section during circumcision, highlight the urgent need to strengthen training of healthcare personnel on basic technical procedures and to standardize practices [17]. Strict oversight of circumcision and awareness of the risks of unregulated practices are essential to prevent such dramatic complications.

The case of urethral meatal stenosis following Type III female genital mutilation illustrates late urological sequelae of FGM [18]. Although Burkina Faso banned FGM in 1996, the practice persists in rural areas, as evidenced by this 4‐year‐old girl. This underscores the importance of intensifying efforts to eradicate harmful traditional practices and ensuring medical follow‐up of victims to detect and treat urological complications early [19].

The presence of four cases of urolithiasis in children highlights a well‐documented problem in sub‐Saharan Africa, including Burkina Faso, where several series report notable hospital frequency of pediatric urolithiasis [20]. Contributing factors include inadequate hydration in the Sahelian climate and diets rich in animal protein and salt. The large stone sizes observed (18–23 mm) reflect prolonged evolution before diagnosis. The lack of endourological facilities in our setting compelled us to use open surgical approaches (cystolithotomy and pyelolithotomy), which are more invasive but allowed complete stone extraction with good outcomes.

Despite the constraints inherent to a resource‐limited setting and the significant diagnostic delays, all patients survived without mortality; however, functional outcomes were variable, as illustrated by the high orchiectomy rate observed in testicular torsion cases (66.7%). The mean hospital stay of 2.8 days and the overall survival rate of 100% demonstrate that adequate therapeutic care is achievable in a regional hospital context, even in the presence of diagnostic delays and limited resources. Nevertheless, the high orchiectomy rate for testicular torsion (66.7%) remains concerning and reflects common challenges in sub‐Saharan Africa: limited access to care and low community awareness.

## 5. Limitations of the Study

This study has several limitations: retrospective design, small sample size (*n* = 28), single‐center nature limiting generalizability, and short‐term follow‐up precluding assessment of late complications.

## 6. Conclusion

Pediatric urological emergencies at the CHR of Dédougou are dominated by trauma and neglected testicular torsions, with major consultation delays leading to high orchiectomy rates. These findings reflect common sub‐Saharan African challenges: limited access to care, low community awareness, and risky traditional practices. Despite material constraints, therapeutic outcomes are favorable. Improving prognosis requires a multidimensional approach: community sensitization, healthcare worker training, and enhanced specialized care access. Multicenter prospective studies are needed to better document this condition in sub‐Saharan Africa.

**Nomenclature**
CHR: Regional Hospital Center
UPJO: Ureteropelvic junction obstruction
FGM: Female genital mutilation

## Author Contributions

Hassami Sawadogo: conceptualization, clinical management, drafting and revision of the manuscript, and corresponding author. Abdoul‐Karim Paré and Abdoul‐Karim Ouattara: literature review and manuscript revision. Fatao Ouédraogo, Békaye Ouédraogo, and Tiéoulé Mamadou Traoré: manuscript revision and critical review. Clotaire Alexis Marie Kiemdiba Donega Yaméogo, Brahima Kirakoya, and Adama Ouattara: supervision and critical revision.

## Funding

No funding was received for this manuscript.

## Disclosure

All authors have read and approved the final version of the manuscript.

## Ethics Statement

This retrospective study was conducted using anonymized patient records. According to institutional policy, formal ethical committee approval was not required for retrospective observational studies. The study was conducted in accordance with the principles of the Declaration of Helsinki. Administrative authorization was obtained from the hospital administration under number 2026‐123/MS/SG/CHR‐DDG/DG/DQ.

## Consent

Written informed consent was obtained from parents or legal guardians for publication of images.

## Conflicts of Interest

The authors declare no conflicts of interest.

## Data Availability

Data sharing is not applicable to this article as no datasets were generated or analyzed during the current study.
